# Senolytics: A Translational Bridge Between Cellular Senescence and Organismal Aging

**DOI:** 10.3389/fcell.2019.00367

**Published:** 2020-01-22

**Authors:** Harikrishnan Thoppil, Karl Riabowol

**Affiliations:** ^1^Arnie Charbonneau Cancer Institute, Department of Biochemistry and Molecular Biology, University of Calgary, Calgary, AB, Canada; ^2^Arnie Charbonneau Cancer Institute, Department of Oncology, University of Calgary, Calgary, AB, Canada

**Keywords:** aging, senescence, lifespan, senolytics, senomorphics

## Abstract

Aging is defined as a progressive decrease in physiological function accompanied by a steady increase in mortality. The antagonistic pleiotropy theory proposes that aging is largely due to the natural selection of genes and pathways that increase fitness and decrease mortality early in life but contribute to deleterious effects and pathologies later in life. Cellular senescence is one such mechanism, which results in a permanent cell cycle arrest that has been described as a mechanism to limit cancer cell growth. However, recent studies have also suggested a dark side of senescence in which a build-up of senescent cells with age leads to increased inflammation due to a senescence-associated secretory phenotype (SASP). This phenotype that includes many cytokines promotes tumorigenesis and can exhaust the pool of immune cells in the body. Studies clearing senescent cells from mice using the p16-based transgene INK-ATTAC have shown that senescent cells can impact both organismal aging and lifespan. Here we discuss these advances that have resulted in the development of a whole new class of compounds known as senolytics, some of which are currently undergoing clinical trials in humans for treating a variety of age-related pathologies such as osteoarthritis.

## Aging

Limited Replicative lifespan was first described by Hayflick and Moorhead (1961), who showed that human diploid fibroblasts have a finite capacity for replication after which they enter a metabolically active but irreversibly arrested proliferative state. Aging has been extensively studied in model organisms such as *Caenorhabditis elegans* and is affected by a variety of factors such as stress, nutrient intake, sex, and gene expression (Moskalev et al., 2015). Several genes have been discovered in these relatively simple model organisms such as DAF-2 (insulin/insulin-like growth factor-1 receptor homolog) which, when mutated results in almost doubling the lifespan of *C. elegans* (Kenyon, 2011). Success in manipulating lifespan in these model organisms ushered in a massive hunt to discover more aging-related genes, mechanisms and therapeutic compounds. At present, The DrugAge database of aging-related drugs lists around 567 distinct chemical perturbagens that can significantly increase lifespan in a subset of non-disease models spanning over 30 species (Barardo et al., 2017). Clinical trials such as The Metformin in Longevity Study (MILES) have been launched recently to assess the anti-aging potential of metformin in delaying age-related ailments in humans (Crandall, 2015; Piskovatska et al., 2019). One of the essential components for studying aging and age-related diseases in humans are biomarkers that are indicative of chronological age (Calimport et al., 2019). Recently it has been shown that DNA methylation patterns show a strong correlation with chronological age and this “epigenetic clock” is effective in predicting all-cause mortality with age (Horvath, 2013). Analyzing cancer tissues with this epigenetic clock, composed of methylation levels from 353 CpGs, indicated that tissues from cancer patients treated with various therapies appeared to be an average of 36 years older compared to the actual chronological age of the patients, while induced pluripotent stem cells (iPSCs) from the same individuals showed resetting of the clock to an epigenetic age of zero. However, the biological mechanisms behind this epigenetic biomarker remain unknown, especially, due to a lack of correlation with gene expression data.

## Senescence

Senescence refers to a state of permanent proliferative arrest characterized by insensitivity to growth factors and mitogens (Kuilman et al., 2010). One of the mechanisms that regulate this insensitivity is dysregulation of normal endocytosis (Wheaton et al., 2001; Rajarajacholan et al., 2013). Senescent cells, were shown to overexpress caveolins, a major component of endocytosis apparatus which prevented their ability to phosphorylate Erk-1/2 phosphorylation post EGF stimulation which was recovered by downregulation via antisense-oligonucleotides. Similar suppression of Erk-1/2 activation was also observed in non-senescent cells post caveolin overexpression (Park, 2002; Park et al., 2002). In cell culture, as observed by Hayflick and Moorhead (1961), a senescence state is achieved upon repeated passaging, and as shown later, it is mainly due to shortening of telomeric DNA found at the end of chromosomes (Harley et al., 1990) that activates an ataxia-telangiectasia mutated (ATM) (Vaziri et al., 1997) and p53-mediated (Atadja et al., 1995) DNA damage response. This was hypothesized first as the end replication problem; as a consequence of semi-conservative DNA replication and later confirmed by Blackburn, Greider, and Szostak (Lundblad and Szostak, 1989; Blackburn, 1991). Senescent cells typically have an enlarged morphology and are most widely detected histochemically by an increased β-galactosidase activity known as senescence-associated β-galactosidase (SA-βGAL) (Itahana et al., 2007), which is correlated to increased autophagy (Young et al., 2009). Other biomarkers of senescence include increased expression of common senescence mediators such as p16, p21, p53, and p47ING1a (Kuilman et al., 2010; Rajarajacholan et al., 2013). However, not all types of senescence result from telomere depletion. For example, up to 50% of mouse embryonic fibroblasts (MEF’s) exhibit a senescence phenotype after a mere five passages resulting from oxygen sensitivity due to ROS-induced DNA damage (Parrinello et al., 2003). These interdependent features of cell cycle withdrawal, macromolecular damage, dysregulated metabolism and an altered senescence-associated secretory phenotype (SASP) have been described as hallmarks of the senescence phenotype, although no markers appear to be universal for all types of senescent cells. Therefore, to ensure the accurate identification of senescent cells it has been recommended by the International Cell Senescence Association that multiple markers be used in a three-step senescence identification protocol (Gorgoulis et al., 2019).

## Mechanisms and Stimuli of Senescence

A wide variety of stimuli affecting multiple molecular pathways are involved in the induction of a senescence based irreversible arrest in a state resembling G_0_ of the cell cycle in mammalian cells (Kuilman et al., 2010; Muñoz-Espín and Serrano, 2014). These pathways are broadly classified into activating either p16 (Alcorta et al., 1996) or p21 (Brown et al., 1997) via the p53 tumor suppresser (Atadja et al., 1995; Vaziri et al., 1997), and these signals converge to produce high levels of active hypophosphorylated Rb (Retinoblastoma) Tumor Suppressor (Chicas et al., 2010; Rajarajacholan et al., 2013). The tumor suppressive nature of cellular senescence is quite evident from the fact that tampering with levels of several of these oncogenes/tumor suppressors can lead to an escape from senescence (Campisi, 1997). Broadly speaking, senescence can be triggered by numerous stresses including DNA-damage, oxidative stress, and oncogene induced stress. DNA-damage induced senescent cells are primarily observed in cancer patients post administration of radiotherapy and chemotherapy (Robles et al., 1999; Gewirtz et al., 2008) and treatment results in accelerated telomere loss (Unryn et al., 2006). Radiotherapy and Chemotherapy not only affect cancer tissue but also induce senescence in surrounding healthy tissue. Several observations have confirmed that exposure to topoisomerase inhibitors such as doxorubicin and etoposide induces senescence in primary diploid human fibroblasts *in vitro* (Robles et al., 1999). Studies have also shown that proteins of the SASP that are released from these surrounding senescent cells can induce an epithelial to mesenchymal transition (EMT) and hence promote malignancy in normally non-aggressive human breast cancer cell lines (Coppé et al., 2008). Another important player in inducing senescence is ING1a from the *IN*hibitor of *G*rowth family of epigenetic regulators. Originally identified in 1996 by subtractive hybridization between cDNAs from normal mammary epithelial cells and transformed breast cancer cells, the *ING* family of genes are evolutionary conserved and ING proteins primarily localize to the nucleus (Garkavtsev et al., 1996). Our lab has shown that the ING1a isoform that is expressed in aging, but not low passage fibroblasts induces senescence when ectopically expressed several-fold faster (24–36 h) compared to other senescence inducing agents such as t-BHP (t-butyl hydroperoxide) and doxorubicin (Rajarajacholan and Riabowol, 2015). We have shown that inhibition of endocytosis is one of the major mechanisms by which ING1a induces senescence (Rajarajacholan et al., 2013; Rajarajacholan and Riabowol, 2015). This occurs via affecting at least three independent members of the Rb pathway including Rb, p16^INK4a^, and p57^Kip2^. ING1a induces expression of Intersectin 2, subsequently altering the stoichiometry of the endocytosis apparatus and blocking signaling by mitogens (Rajarajacholan et al., 2013). This represents a novel mechanism of senescence induction that is independent of telomere attrition and DNA damage signaling.

## Cellular Senescence and Organismal Aging

**FIGURE 1:**
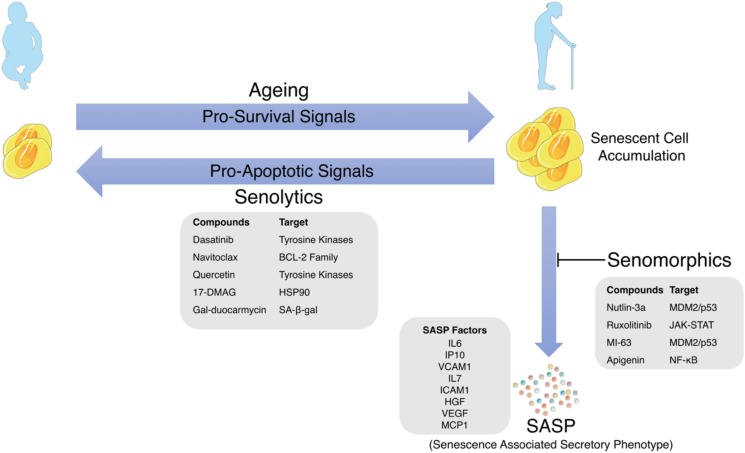
Schematic representation of the link between cellular senescence and aging with examples of SASP Factors, Senolytics and Senomorphics and their targets of action.

One of the hallmarks of mammalian aging is the accumulation of irreversibly arrested senescent cells in various tissues (Figure 1). In a 2006 primate study, it was observed that senescent cells, as estimated by ATM activation do accumulate and can reach over 15% of the total cell population in aged individuals (Herbig et al., 2006). In contrast to the vast majority of *in vitro* studies, this was one of the first studies showing a clear association between aging and the accumulation of senescent cells *in vivo*. Although this established a strong correlation, efforts were underway to establish causation between the accumulation of senescent cells and aging *in vivo*. In 2011, the Kirkland lab showed that removing p16^Ink4a^ positive senescent cells delayed age-related disorders and increased healthspan in a BubR1 progeroid accelerated model of aging mice (Baker et al., 2011). This was accomplished using a novel transgene composed of a p16^Ink4a^ promoter driving a FKBP–Caspase-8 fusion protein, which is selectively expressed in senescent cells and can be activated by the synthetic drug, AP20187. These transgenic mice showed delayed onset of sarcopenia, cataracts, significantly better exercise test scores and reduction in other age-related pathologies. Later studies confirmed the beneficial effects of senescent cell removal in wild type mice that showed increased median lifespan, delayed tumorigenesis and attenuated age-related multi-organ deterioration (Baker et al., 2016). Removal of senescent cells in mice has also been shown to attenuate markers of age-associated neurodegenerative diseases such as tau hyperphosphorylation and neurofibrillary tangle deposition (Bussian et al., 2018). Since it is likely that even a small percentage of senescent cells can promote substantial deleterious effects, there may be natural mechanisms that clear these senescent cells *in vivo* to maintain overall fitness. One primary response to p53 activation in murine liver carcinoma was found to be the accumulation of senescent cells, which were subsequently shown to be cleared by the innate immune response (Xue et al., 2011). NK cells were also found to eliminate senescent cells *in vivo* by perforin (Prf) mediated granule exocytosis (Sagiv et al., 2013) and Prf1^–/–^ mice showed a 4X accumulation of senescent cells and a 21% decrease in median lifespan, which was alleviated by senescent cell clearance (Ovadya et al., 2018).

Further evidence regarding the connection between cellular senescence and organismal aging comes from accelerated aging syndromes such as HGPS (Hutchinson-Gilford progeria syndrome). A higher proportion of HGPS fibroblasts are SA-β-Gal positive compared to fibroblasts from normal, age-matched controls, and they can be returned to a normal replicative state by exogenous expression of the TERT subunit of telomerase (Benson et al., 2010). Similarly, expressing progerin, the mutant lamin-A protein known to cause HGPS, in primary cells obtained from healthy individuals result in expression of senescent markers (Cao et al., 2011). These results suggest that progerin-induced telomere dysfunction may result in premature cellular senescence leading to the accelerated aging symptoms observed in HGPS patients.

## Senolytics and Senomorphics

Substantial evidence in the last decade connecting senescent cell accumulation, age-related ailments, and roles in lifespan and healthspan fueled the search for therapeutic compounds that could selectively target senescent cells. A transcriptomic analysis between senescent cells and proliferating cells showed increased expression of pro-survival/anti-apoptotic genes such as Bcl-xL (B-cell lymphoma-extra-large) a member of the Bcl-2 family of proteins that regulates programmed cell death by blocking caspase activation (Zhu et al., 2016). This provided evidence to support the observation that senescent cells accumulate with age by being resistant to a variety of stresses that would normally induce apoptosis (Wang, 1995). Consistent with this idea, siRNAs to reduce Bcl-xL expression selectively reduced survival and viability in senescent cells while not affecting proliferating cells (Zhu et al., 2015). Quercetin and Dasatinib were obtained as hits from a drug screen based on these observations (Hickson et al., 2019). Quercetin: a flavonoid and Dasatinib: an anticancer agent, are known inhibitors of a variety of tyrosine kinases (Huang et al., 1999; Montero et al., 2011). These compounds form one of the first discovered members of the senolytic class of drugs that selectively induce apoptosis in senescent cells. Four years after their initial identification as candidate senolytics, a Dasatinib + Quercetin combination was reported to decrease the senescent cell burden in humans as part of a Phase-1 clinical trial in diabetic kidney disease patients (Hickson et al., 2019). This 2019 study was the first peer-reviewed study to demonstrate the efficacy of senolytics to decrease senescent cell burden in humans. This was carried out after an initial pilot study in early 2019 in 14 idiopathic pulmonary fibrosis (IPF) patients was completed to evaluate the feasibility of implementing a senolytic treatment (Justice et al., 2019). What now remains to be determined is whether future clinical trials will demonstrate any positive medical outcomes resulting from decreased senescent cell burden in diabetes and other age-associated ailments.

Another senolytic strategy is the inhibition of the interaction between Mouse Double Minute 2 (MDM2) and p53 that usually results in the ubiquitination and proteasomal degradation of p53 (Nag et al., 2013; Wade et al., 2013). The MDM2 protein acts as an E3 ubiquitin ligase and facilitates p53 degradation. *MDM2* contains two promoters: P1 and P2. P1 is constitutively active at low levels while the P2 promoter has p53 binding sites and acts as a negative regulator of p53 (Hollerer et al., 2019). The MDM2 antagonists Nutlin-3a and MI-63 have been shown to increase p53 levels and attenuate the secretory phenotype of senescent cells (SASP) (Wiley et al., 2018). These represent a new class of compounds known as “Senomorphics,” which attenuate the expression of cytokines such as IL-6 (Interleukin-6) that make up the SASP. UBX0101, a compound from the Unity Biotechnology pipeline is another promising senolytic that inhibits the MDM2/p53 interaction and is currently undergoing Phase 1 clinical trial against osteoarthritis. UBX0101 administration was reported to decrease classical osteoarthritis-related phenotypes such as cartilage erosion and joint pain in mice by p53-mediated clearing of senescent cells. This study suggested that senescent cells might promote osteoarthritis (Jeon et al., 2017), another medically relevant age-related pathology.

Heat Shock Protein 90 (HSP90) might represent another target for senolysis. This protein is one member of a large family of chaperones that help other proteins to fold and refold after cell stress and is indirectly involved in a wide variety of cellular processes such as DNA repair, heat stress response and neurodegenerative pathologies (Schopf et al., 2017). While HSP90 levels remain relatively similar in senescent and non-senescent cells, AKT (Protein Kinase B), which is a downstream effector of HSP90 is highly expressed in senescent cells. Inhibiting HSP90 destabilizes AKT and results in increased apoptosis (Karkoulis et al., 2013). HSP90 inhibitors such as 17-DMAG were discovered through a screen for senolytic compounds based on SA-βGAL positive cell count as an endpoint. 17-DMAG was further shown to be senolytic *in vivo* by decreasing p16 positive cells and extending healthspan in Ercc1^–/Δ^ mice (Fuhrmann-Stroissnigg et al., 2017).

## The Next Generation of Senolytics

Since senescence is a complex phenotype involving multiple pathways and proteins, it is unlikely that a single senolytic compound targeting a single protein will be able to eliminate all types of senescent cells. For example, Quercetin is more selective against senescent human endothelial cells, while Dasatinib is far more effective against senescent human primary preadipocytes (Hickson et al., 2019). Similarly, while Navitoclax, a senolytic agent targeting the Bcl-2 family of proteins is selective against senescent human umbilical vein epithelial cells (HUVECs) and IMR90 human lung fibroblasts, it is not particularly effective against primary human preadipocytes (Zhu et al., 2016). Senolytic compounds that target senescent cells more broadly while maintaining low cytotoxicity by increased selectivity solely for cells that are truly senescent are therefore of great interest! Elevated activity of the lysosomal β-galactosidase, which is a nearly universal characteristic of senescent cells (Lee et al., 2006) and has been exploited as a senescence biomarker can also be utilized for drug selectivity against a wide range of senescent cells. One example is a drug delivery system utilizing encapsulation of cytotoxic drugs with galacto-oligosaccharides (Muñoz-Espín et al., 2018). These capsules that can be loaded with a variety of cytotoxic compounds, take advantage of the high senescent cell lysosomal β-galactosidase activity to preferentially release their cargo within senescent cells. A very recent study also used a similar strategy involving galactose-modified prodrug. Here, preferential processing of galactose-modified duocarmycin (GMD), a cytotoxic conjugate, was used to eliminate a wide variety of senescent cell types (Guerrero et al., 2019). Targeting cell surface proteins enriched in senescent cells such as DPP4 (dipeptidyl peptidase 4) can also be used to enhance selectivity (Kim et al., 2017). Another cell surface protein, the CD9 receptor has also been used to target senescent cells and their secretory phenotype with anti-CD9 monoclonal antibody encapsulated, lactose-wrapped nanoparticles, loaded with rapamycin as the payload (Thapa et al., 2017). These nanoparticles have dual selectivity toward senescent cells based on both increased CD9 receptors on the cell surface and increased lysosomal β-galactosidase expression.

## Conclusion

The role of cellular senescence in organismal aging has been well established in the past two decades in a variety of organisms (Fick et al., 2012; Heidinger et al., 2012; Muñoz-Lorente et al., 2019). From initial thoughts indicating that senescence served primarily as an anti-cancer mechanism, we note the emerging view of senescence also acting as a driving force behind a wide variety of age-related pathologies (Fernandes et al., 2016). More recent studies in the last decade, which resulted in the development of transgenes and chemical perturbagens, have finally given us the tools to selectively manipulate the quantity of senescent cells *in vivo*. Thorough characterization of their deleterious secretory phenotype, that varies in different cell types, continues to improve our understanding of how they contribute to the wide variety of age-related diseases observed in our populations. Finally, as the first Senolytics enter clinical trials, we are on the cusp of establishing a translational bridge between cellular senescence and organismal aging (Muñoz-Espín and Serrano, 2014; Calimport et al., 2019; Gorgoulis et al., 2019) that may help ameliorate the burden of many age-related pathologies.

## Author Contributions

All authors listed have made a substantial, direct and intellectual contribution to the work, and approved it for publication.

## Conflict of Interest

The authors declare that the research was conducted in the absence of any commercial or financial relationships that could be construed as a potential conflict of interest.

## References

[B1] Alcorta D. A. Xiong Y. Phelps D. Hannon G. Beach D. Barrett J. C. (1996). Involvement of the cyclin-dependent kinase inhibitor p16 (INK4a) in replicative senescence of normal human fibroblasts. *Proc. Natl. Acad. Sci. U.S.A.* 93 13742–13747. 10.1073/pnas.93.24.13742 8943005 PMC19411

[B2] Atadja P. Wong H. Garkavtsev I. Veillette C. Riabowol K. (1995). Increased activity of p53 in senescing fibroblasts. *Proc. Natl. Acad. Sci. U.S.A.* 92 8348–8352. 10.1073/pnas.92.18.8348 7667293 PMC41154

[B3] Baker D. J. Childs B. G. Durik M. Wijers M. E. Sieben C. J. Zhong J. (2016). Naturally occurring p16 Ink4a-positive cells shorten healthy lifespan. *Nature* 530 184–189. 10.1038/nature16932 26840489 PMC4845101

[B4] Baker D. J. Wijshake T. Tchkonia T. Lebrasseur N. K. Childs B. G. Van De Sluis B. (2011). Clearance of p16 Ink4a-positive senescent cells delays aging-associated disorders. *Nature* 479 232–236. 10.1038/nature10600 22048312 PMC3468323

[B5] Barardo D. Thornton D. Thoppil H. Walsh M. Sharifi S. Ferreira S. (2017). The drugage database of aging-related drugs. *Aging Cell* 16 594–597. 10.1111/acel.12585 28299908 PMC5418190

[B6] Benson E. K. Lee S. W. Aaronson S. A. (2010). Role of progerin-induced telomere dysfunction in HGPS premature cellular senescence. *J. Cell Sci.* 123 2605–2612. 10.1242/jcs.067306 20605919 PMC2908049

[B7] Blackburn E. H. (1991). Structure and function of telomeres. *Nature* 350 569–573. 10.1038/350569a0 1708110

[B8] Brown J. P. Wei W. Sedivy J. M. (1997). Bypass of senescenoe after disruption of p21(CIP1)/(WAF1) gene in normal diploid human fibroblasts. *Science* 277 831–834. 10.1126/science.277.5327.831 9242615

[B9] Bussian T. J. Aziz A. Meyer C. F. Swenson B. L. van Deursen J. M. Baker D. J. (2018). Clearance of senescent glial cells prevents tau-dependent pathology and cognitive decline. *Nature* 562 578–582. 10.1038/s41586-018-0543-y 30232451 PMC6206507

[B10] Calimport S. R. G. Bentley B. L. Stewart C. E. Pawelec G. Scuteri A. Vinciguerra M. (2019). To help aging populations, classify organismal senescence. *Science* 366 576–578. 10.1126/science.aay7319 31672885 PMC7193988

[B11] Campisi J. (1997). The biology of replicative senescence. *Eur. J. Cancer Part A* 33 703–709. 10.1016/S0959-8049(96)00058-5 9282108

[B12] Cao K. Blair C. D. Faddah D. A. Kieckhaefer J. E. Olive M. Erdos M. R. (2011). Progerin and telomere dysfunction collaborate to trigger cellular senescence in normal human fibroblasts. *J. Clin. Invest.* 121 2833–2844. 10.1172/JCI43578 21670498 PMC3223819

[B13] Chicas A. Wang X. Zhang C. McCurrach M. Zhao Z. Mert O. (2010). Dissecting the unique role of the retinoblastoma tumor suppressor during cellular senescence. *Cancer Cell* 17 376–387. 10.1016/j.ccr.2010.01.023 20385362 PMC2889489

[B14] Coppé J. P. Patil C. K. Rodier F. Sun Y. Muñoz D. P. Goldstein J. (2008). Senescence-associated secretory phenotypes reveal cell-nonautonomous functions of oncogenic RAS and the p53 tumor suppressor. *PLoS Biol.* 6: 2853–68. 10.1371/journal.pbio.0060301 19053174 PMC2592359

[B15] Crandall J. (2015). *Metformin in Longevity Study (MILES).* Available at: https://clinicaltrials.gov/ct2/show/NCT02432287 (accessed May 31, 2018).

[B16] Fernandes M. Wan C. Tacutu R. Barardo D. Rajput A. Wang J. (2016). Systematic analysis of the gerontome reveals links between aging and age-related diseases. *Hum. Mol. Genet.* 25 4804–4818. 10.1093/hmg/ddw307 28175300 PMC5418736

[B17] Fick L. J. Fick G. H. Li Z. Cao E. Bao B. Heffelfinger D. (2012). Telomere length correlates with life span of dog breeds. *Cell Rep.* 2 1530–1536. 10.1016/j.celrep.2012.11.021 23260664

[B18] Fuhrmann-Stroissnigg H. Ling Y. Y. Zhao J. McGowan S. J. Zhu Y. Brooks R. W. (2017). Identification of HSP90 inhibitors as a novel class of senolytics. *Nat. Commun.* 8:422. 10.1038/s41467-017-00314-z 28871086 PMC5583353

[B19] Garkavtsev I. Kazarov A. Gudkov A. Riabowol K. (1996). Suppression of the novel growth inhibitor p33(ING1) promotes neoplastic transformation. *Nat. Genet.* 14 415–420. 10.1038/ng1296-415 8944021

[B20] Gewirtz D. A. Holt S. E. Elmore L. W. (2008). Accelerated senescence: An emerging role in tumor cell response to chemotherapy and radiation. *Biochem. Pharmacol.* 76 947–957. 10.1016/j.bcp.2008.06.024 18657518

[B21] Gorgoulis V. Adams P. D. Alimonti A. Bennett D. C. Bischof O. Bishop C. (2019). Cellular senescence: defining a path forward. *Cell* 179 813–827. 10.1016/j.cell.2019.10.005 31675495

[B22] Guerrero A. Guiho R. Herranz N. Uren A. Withers D. J. Martínez-Barbera J. P. (2019). Galactose-modified duocarmycin prodrugs as senolytics. *bioRxiv* [Preprint] 10.1101/746669 PMC7189988 32175667

[B23] Harley C. B. Futcher A. B. Greider C. W. (1990). Telomeres shorten during aging of human fibroblasts. *Nature* 345 458–460. 10.1038/345458a0 2342578

[B24] Hayflick L. Moorhead P. S. (1961). The serial cultivation of human diploid cell strains. *Exp. Cell Res.* 25 585–621. 10.1016/0014-4827(61)90192-6 13905658

[B25] Heidinger B. J. Blount J. D. Boner W. Griffiths K. Metcalfe N. B. Monaghan P. (2012). Telomere length in early life predicts lifespan. *Proc. Natl. Acad. Sci. U.S.A.* 109 1743–1748. 10.1073/pnas.1113306109 22232671 PMC3277142

[B26] Herbig U. Ferreira M. Condel L. Carey D. Sedivy J. M. (2006). Cellular senescence in aging primates. *Science* 311:1257. 10.1126/science.1122446 16456035

[B27] Hickson L. T. J. Langhi Prata L. G. P. Bobart S. A. Evans T. K. Giorgadze N. Hashmi S. K. (2019). Senolytics decrease senescent cells in humans: preliminary report from a clinical trial of dasatinib plus quercetin in individuals with diabetic kidney disease. *EBioMedicine* 47 446–456. 10.1016/j.ebiom.2019.08.069 31542391 PMC6796530

[B28] Hollerer I. Barker J. C. Jorgensen V. Tresenrider A. Dugast-Darzacq C. Chan L. Y. (2019). Evidence for an integrated gene repression mechanism based on mRNA isoform toggling in human cells. *G3* 9 1045–1053. 10.1534/g3.118.200802 30723103 PMC6469420

[B29] Horvath S. (2013). DNA methylation age of human tissues and cell types. *Genome Biol.* 14:R115. 10.1186/gb-2013-14-10-r115 24138928 PMC4015143

[B30] Huang Y. T. Hwang J. J. Lee P. P. Ke F. C. Huang J. H. Huang C. J. (1999). Effects of luteolin and quercetin, inhibitors of tyrosine kinase, on cell growth and metastasis-associated properties in A431 cells overexpressing epidermal growth factor receptor. *Br. J. Pharmacol.* 128 999–1010. 10.1038/sj.bjp.0702879 10556937 PMC1571723

[B31] Itahana K. Campisi J. Dimri G. P. (2007). Methods to detect biomarkers of cellular senescence. *Methods Mol. Biol.* 371 21–31. 10.1007/978-1-59745-361-5_3 17634571

[B32] Jeon O. H. Kim C. Laberge R. M. Demaria M. Rathod S. Vasserot A. P. (2017). Local clearance of senescent cells attenuates the development of post-traumatic osteoarthritis and creates a pro-regenerative environment. *Nat. Med.* 23 775–781. 10.1038/nm.4324 28436958 PMC5785239

[B33] Justice J. N. Nambiar A. M. Tchkonia T. LeBrasseur N. K. Pascual R. Hashmi S. K. (2019). Senolytics in idiopathic pulmonary fibrosis: Results from a first-in-human, open-label, pilot study. *EBioMedicine* 40 554–563. 10.1016/j.ebiom.2018.12.052 30616998 PMC6412088

[B34] Karkoulis P. K. Stravopodis D. J. Konstantakou E. G. Voutsinas G. E. (2013). Targeted inhibition of heat shock protein 90 disrupts multiple oncogenic signaling pathways, thus inducing cell cycle arrest and programmed cell death in human urinary bladder cancer cell lines. *Cancer Cell Int.* 13:11. 10.1186/1475-2867-13-11 23394616 PMC3583703

[B35] Kenyon C. (2011). The first long-lived mutants: Discovery of the insulin/IGF-1 pathway for aging. *Philos. Trans. R. Soc. B Biol. Sci.* 366 9–16. 10.1098/rstb.2010.0276 21115525 PMC3001308

[B36] Kim K. M. Noh J. H. Bodogai M. Martindale J. L. Yang X. Indig F. E. (2017). Identification of senescent cell surface targetable protein DPP4. *Genes Dev.* 31 1529–1534. 10.1101/gad.302570.117 28877934 PMC5630018

[B37] Kuilman T. Michaloglou C. Mooi W. J. Peeper D. S. (2010). The essence of senescence. *Genes Dev.* 24 2463–2479. 10.1101/gad.1971610 21078816 PMC2975923

[B38] Lee B. Y. Han J. A. Im J. S. Morrone A. Johung K. Goodwin E. C. (2006). Senescence-associated β-galactosidase is lysosomal β-galactosidase. *Aging Cell* 5 187–195. 10.1111/j.1474-9726.2006.00199.x 16626397

[B39] Lundblad V. Szostak J. W. (1989). A mutant with a defect in telomere elongation leads to senescence in yeast. *Cell* 57 633–643. 10.1016/0092-8674(89)90132-3 2655926

[B40] Montero J. C. Seoane S. Ocaña A. Pandiella A. (2011). Inhibition of Src family kinases and receptor tyrosine kinases by dasatinib: Possible combinations in solid tumors. *Clin. Cancer Res.* 17 5546–5552. 10.1158/1078-0432.CCR-10-2616 21670084

[B41] Moskalev A. Chernyagina E. de Magalhães J. P. Barardo D. Thoppil H. Shaposhnikov M. (2015). Geroprotectors.org: a new, structured and curated database of current therapeutic interventions in aging and age-related disease. *Aging* 7 616–628. 10.18632/aging.100799 26342919 PMC4600621

[B42] Muñoz-Espín D. Serrano M. (2014). Cellular senescence: from physiology to pathology. *Nat. Rev. Mol. Cell Biol.* 15 482–496. 10.1038/nrm3823 24954210

[B43] Muñoz-Lorente M. A. Cano-Martin A. C. Blasco M. A. (2019). Mice with hyper-long telomeres show less metabolic aging and longer lifespans. *Nat. Commun.* 10:4723. 10.1038/s41467-019-12664-x 31624261 PMC6797762

[B44] Muñoz-Espín D. Rovira M. Galiana I. Giménez C. Lozano-Torres B. Paez-Ribes M. (2018). A versatile drug delivery system targeting senescent cells. *EMBO Mol. Med.* 10: e9355. 10.15252/emmm.201809355 30012580 PMC6127887

[B45] Nag S. Qin J. Srivenugopal K. S. Wang M. Zhang R. (2013). The MDM2-p53 pathway revisited. *J. Biomed. Res.* 27 254–271. 10.7555/JBR.27.20130030 23885265 PMC3721034

[B46] Ovadya Y. Landsberger T. Leins H. Vadai E. Gal H. Biran A. (2018). Impaired immune surveillance accelerates accumulation of senescent cells and aging. *Nat. Commun.* 9:5435. 10.1038/s41467-018-07825-3 30575733 PMC6303397

[B47] Park S. C. (2002). Functional recovery of senescent cells through restoration of receptor-mediated endocytosis. *Mech. Aging Dev.* 123 917–926. 10.1016/S0047-6374(02)00029-5 12044940

[B48] Park S. C. Park J. S. Park W. Y. Cho K. A. Ahn J. S. Jang I. S. (2002). Down-regulation of receptor-mediated endocytosis is reponsible for senescence-associated hyporesponsiveness. *Ann. N. Y. Acad. Sci.* 959 45–49. 10.1111/j.1749-6632.2002.tb02081.x 11976184

[B49] Parrinello S. Samper E. Krtolica A. Goldstein J. Melov S. Campisi J. (2003). Oxygen sensitivity severely limits the replicative lifespan of murine fibroblasts. *Nat. Cell Biol.* 5 741–747. 10.1038/ncb1024 12855956 PMC4940195

[B50] Piskovatska V. Stefanyshyn N. Storey K. B. Vaiserman A. M. Lushchak O. (2019). Metformin as a geroprotector: experimental and clinical evidence. *Biogerontology* 20 33–48. 10.1007/s10522-018-9773-5 30255224

[B51] Rajarajacholan U. K. Riabowol K. (2015). Aging with ING: a comparative study of different forms of stress induced premature senescence. *Oncotarget* 6 34118–34127. 10.18632/oncotarget.5947 26439691 PMC4741440

[B52] Rajarajacholan U. K. Thalappilly S. Riabowol K. (2013). The ING1a tumor suppressor regulates endocytosis to induce cellular senescence via the Rb-E2F pathway. *PLoS Biol.* 11: e1001502. 10.1371/journal.pbio.1001502 23472054 PMC3589274

[B53] Robles S. J. Buehler P. W. Negrusz A. Adami G. R. (1999). Permanent cell cycle arrest in asynchronously proliferating normal human fibroblasts treated with doxorubicin or etoposide but not camptothecin. *Biochem. Pharmacol.* 58 675–685. 10.1016/S0006-2952(99)00127-6 10413306

[B54] Sagiv A. Biran A. Yon M. Simon J. Lowe S. W. Krizhanovsky V. (2013). Granule exocytosis mediates immune surveillance of senescent cells. *Oncogene* 32 1971–1977. 10.1038/onc.2012.206 22751116 PMC3630483

[B55] Schopf F. H. Biebl M. M. Buchner J. (2017). The HSP90 chaperone machinery. *Nat. Rev. Mol. Cell Biol.* 18 345–360. 10.1038/nrm.2017.20 28429788

[B56] Thapa R. K. Nguyen H. T. Jeong J. H. Kim J. R. Choi H. G. Yong C. S. (2017). Progressive slowdown/prevention of cellular senescence by CD9-targeted delivery of rapamycin using lactose-wrapped calcium carbonate nanoparticles. *Sci. Rep.* 7:43299. 10.1038/srep43299 28393891 PMC5385881

[B57] Unryn B. M. Hao D. Glück S. Riabowol K. T. (2006). Acceleration of telomere loss by chemotherapy is greater in older patients with locally advanced head and neck cancer. *Clin. Cancer Res.* 12 6345–6350. 10.1158/1078-0432.CCR-06-0486 17085644

[B58] Vaziri H. West M. D. Allsopp R. C. Davison T. S. Wu Y. S. Arrowsmith C. H. (1997). ATM-dependent telomere loss in aging human diploid fibroblasts and DNA damage lead to the post-translational activation of p53 protein involving poly(ADP-ribose) polymerase. *EMBO J.* 16 6018–6033. 10.1093/emboj/16.19.6018 9312059 PMC1170232

[B59] Wade M. Li Y. C. Wahl G. M. (2013). MDM2, MDMX and p53 in oncogenesis and cancer therapy. *Nat. Rev. Cancer* 13 83–96. 10.1038/nrc3430 23303139 PMC4161369

[B60] Wang E. (1995). Senescent human fibroblasts resist programmed cell death, and failure to suppress bell is involved. *Cancer Res.* 55 2284–2292. 7757977

[B61] Wheaton K. Sampsel K. Boisvert F. M. Davy A. Robbins S. Riabowol K. (2001). Loss of functional caveolae during senescence of human fibroblasts. *J. Cell. Physiol.* 187 226–235. 10.1002/jcp.1071 11268002

[B62] Wiley C. D. Schaum N. Alimirah F. Lopez-Dominguez J. A. Orjalo A. V. Scott G. (2018). Small-molecule MDM2 antagonists attenuate the senescence-associated secretory phenotype. *Sci. Rep.* 8:2410. 10.1038/s41598-018-20000-4 29402901 PMC5799282

[B63] Xue W. Zender L. Miething C. Dickins R. A. Hernando E. Krizhanovsky V. (2011). Senescence and tumour clearance is triggered by p53 restoration in murine liver carcinomas (Nature (2007) 445 (656-660)). *Nature* 473:544 10.1038/nature09909 PMC4601097 17251933

[B64] Young A. R. J. Narita M. Ferreira M. Kirschner K. Sadaie M. Darot J. F. J. (2009). Autophagy mediates the mitotic senescence transition. *Genes Dev.* 23 798–803. 10.1101/gad.519709 19279323 PMC2666340

[B65] Zhu Y. Tchkonia T. Fuhrmann-Stroissnigg H. Dai H. M. Ling Y. Y. Stout M. B. (2016). Identification of a novel senolytic agent, navitoclax, targeting the Bcl-2 family of anti-apoptotic factors. *Aging Cell* 15 428–435. 10.1111/acel.12445 26711051 PMC4854923

[B66] Zhu Y. Tchkonia T. Pirtskhalava T. Gower A. C. Ding H. Giorgadze N. (2015). The Achilles’ heel of senescent cells: from transcriptome to senolytic drugs. *Aging Cell* 14 644–658. 10.1111/acel.12344 25754370 PMC4531078

